# Supplementary material for: Splice-Junction-Based Mapping of Alternative Isoforms in the Human Proteome
Source: Cell Rep. Author manuscript; Available in PMC 2020 Jan 15. (PMC6961840; doi:10.1016/j.celrep.2019.11.026)
Supplement: 2 [file NIHMS1546469-supplement-2.zip › DF1/PXD010154/Liver/Liver_46_GMPR2_VTQQVNPIFSEAGSHK.pdf]

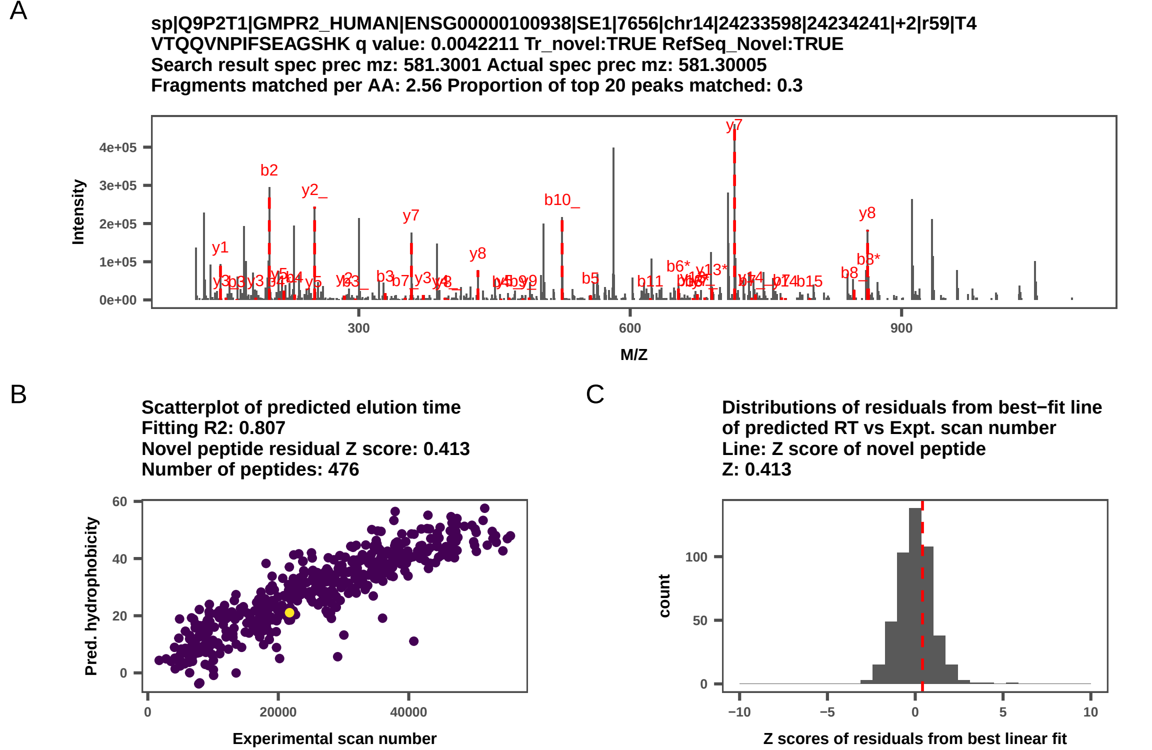
A
sp|Q9P2T1|GMPR2_HUMAN|ENSG00000100938|SE1|7656|chr14|24233598|24234241|+2|r59|T4
VTQQVNPIFSEAGSHK q value: 0.0042211 Tr_novel:TRUE RefSeq_Novel:TRUE
Search result spec prec mz: 581.3001 Actual spec prec mz: 581.30005
Fragments matched per AA: 2.56 Proportion of top 20 peaks matched: 0.3
Intensity
4e+05
3e+05
2e+05
1e+05
0e+00
y7
b2
y2_
b10_
y7
y8
y1
y8
b6*
y13*
b8*
b8_
b5
b11
b15
300
600
900
M/Z
B
Scatterplot of predicted elution time
Fitting R2: 0.807
Novel peptide residual Z score: 0.413
Number of peptides: 476
Pred. hydrophobicity
60
40
20
0
0
20000
40000
Experimental scan number
C
Distributions of residuals from best−fit line
of predicted RT vs Expt. scan number
Line: Z score of novel peptide
Z: 0.413
count
100
50
0
−10
−5
0
5
10
Z scores of residuals from best linear fit
